# State of Nursing in Brazil

**DOI:** 10.1590/1518-8345.0000.3404

**Published:** 2020-12-09

**Authors:** Ana Paula Cavalcante de Oliveira, Carla Aparecida Arena Ventura, Francisca Valda da Silva, Hélio Angotti, Isabel Amelia Costa Mendes, Kleyde Ventura de Souza, Mayra Isabel Correia Pinheiro, Manoel Carlos Neri da Silva, Mónica Padilla, Nádia Mattos Ramalho, Wagner Villas Boas de Souza

**Affiliations:** 1 Organização Pan-Americana da Saúde/Organização Mundial da Saúde (OPAS/OMS), Unidade Técnica de Capacidades Humanas para a Saúde, Brasília, DF, Brazil.; 2 Universidade de São Paulo, Escola de Enfermagem de Ribeirão Preto, PAHO/WHO Collaborating Centre for Nursing Research Development, Ribeirão Preto, SP, Brazil.; 3 Associação Brasileira de Enfermagem Nacional, Brasília, DF, Brazil.; 4 Ministério da Saúde, Secretaria de Gestão do Trabalho e da Educação na Saúde, Brasília, DF, Brazil.; 5 Grupo de Trabalho Campanha Nursing Now Brazil.; 6 Associação Brasileira de Obstetrizes e Enfermeiros Obstetras Nacional, Rio de Janeiro, RJ, Brazil.; 7 Conselho Federal de Enfermagem, Brasília, DF, Brazil.; 8 Ministério da Educação, Secretaria de Educação Superior, Brasília, DF, Brazil.


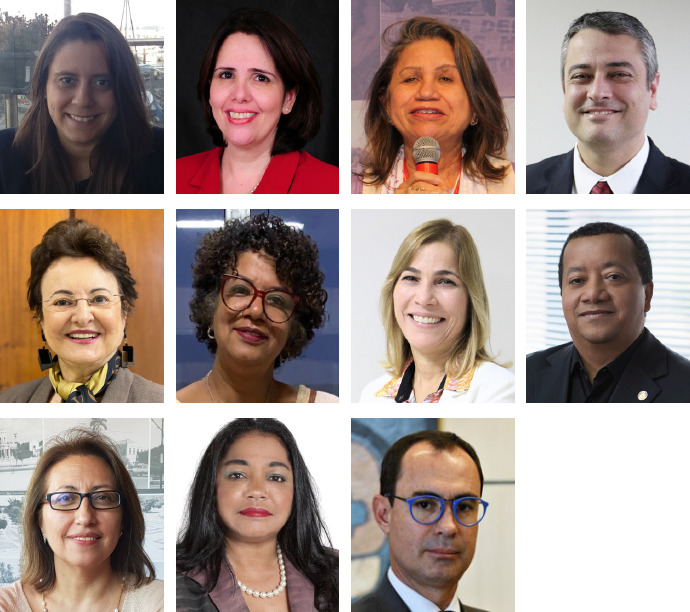


The nursing working force is essential in the provision of integrated care centered on people, playing a fundamental role in the performance of health priorities and in accomplishing the Sustainable Development Goals (SDGs). The importance of its performance has been recognized and still more decisive given the challenges faced worldwide in an international emergency scenario such as the COVID-19 pandemic. In the scope of the celebrations of the International Year of Nursing and Obstetrics (2020)^(^ 1 ^)^ two reports are being organized with contributions from representatives of the World Health Organization (WHO) member countries and their regions: “State of the World's Nursing 2020: investing in education, jobs and leadership”^(^ 2 ^)^, organized by the WHO along with the International Council of Nurses (ICN) and the Nursing Now campaign, and the “State of the World's Obstetrics”, with launch scheduled for 2021, organized by the WHO, the International Confederation of Midwives (ICM) and the ICN.

By means of collaborative work, the Brazilian Nursing Association (*Associação Brasileira de Enfermagem*, ABEn), the Brazilian Association of Obstetricians and Obstetric Nurses (*Associação Brasileira de Obstetrizes e Enfermeiros Obstetras*, Abenfo), the Federal Nursing Council (*Conselho Federal de Enfermagem*, Cofen), the Ministry of Education, the Ministry of Health, the Pan-American Health Organization (PAHO/WHO) Collaborative Center for Development and Research in Nursing of the Nursing School of Ribeirão Preto at the University of São Paulo, the Nursing Now workgroup in Brazil, and the Pan-American Health Organization (PAHO/WHO) Technical Unit for Health Human Capacities representation in Brazil, made a commitment to contribute to the organization of these reports and to draw up an infographic highlighting the peculiarities of the Nursing Profile in Brazil, “Picture of Brazil's Nursing”, launched in June 2020^(^ 3 ^)^. The analysis of the Brazilian data highlighted the need for identification, outlining, and implementation of public policies to face the challenges of nursing in the country.

Nursing workers add up to 27.9 million professionals, with more than 80% in countries that account for half of the world population. The region of the Americas adds up to 8.4 million professionals (approximately 30% of the world total), with 87% located in Brazil, Canada, and the United States of America, which represent approximately 57% of the region's population. Between 2013 and 2018, there was an increase of 4.7 million professionals in global Nursing stock. However, an estimated shortage of 5.9 million professionals persists in 2018^(^ 2 ^)^. In Brazil, data demonstrate a 39% increase in the number of professionals in the same period, totaling 2,119,620 professionals qualified for professional practice in 2018, and an increase projection of approximately 51% for 2030^(^ 2 ^-^ 3 ^)^.

Nursing constitutes the largest occupational group in the health sector, with approximately 59% of the world's health workforce and 56% in the Americas^(^ 2 ^)^. In Brazil, Nursing (composed of nurses and nursing technicians and assistants) accounted for almost 70% of the professionals (17% nurses, 53% nursing assistants and technicians), followed by physicians (15.70%), dentists (9%), pharmacists (4,9%), and obstetricians (0.2%)* ^,^ ** ^(^ 3 ^)^.

Regarding density, the data demonstrate a significant variation between the regions, with a global mean of 36.9 of nursing professionals per 10,000 inhabitants. On the one hand, the Region of the Americas has 83.4 professionals, while the African Region had a mean density of 8.7 professionals^(^ 2 ^)^. The concerning inequality in distribution persists among countries and also among the Brazilian federative units. In Brazil, a density of 101.4 nursing professionals per 10,000 inhabitants was verified in 2018, with the state of Alagoas presenting a density of 73.69 and the Federal District with 163.60, for example. When analyzing the density between the distribution of nurses, the disparity is even greater. In Brazil, there are 24.54 nurses per 10,000 inhabitants; in states like Pará, data demonstrate a density of 14.13 nurses and, in the Federal District, there are 43.39 nurses (much higher than national density)^(^ 3 ^)^.

Using the 2008 Uniform Occupation Classification (*Classificação Uniforme de Ocupações*, CIUO-08) to enable comparisons, it was possible to identify that 19.3 million, almost 69% of the nursing professionals in the world, are nurses with a higher education degree (“professional nurses”), 6 million (22%) are mid-level nursing professionals (“associated nurses”), and 2.6 million (9%) were not included in any of these two groups, which can indicate possible aligning difficulties between the national data systems and/or the national classifications of occupations and the CIUO^(^ 2 ^)^. In Brazil, the opposite of the world scene stands out, since that nursing is mostly composed of mid-level professionals (76% of nursing technicians and auxiliaries) and 24% of nurses (higher education)^(^ 3 ^)^. In the Americas, most of the professionals also are mid-level; however, in a smaller proportion than that of Brazil: 59% of mid-level, 36% of higher level, and 5% not classified^(^ 2 ^)^.

With regard to the global distribution by age groups, the nursing workforce is relatively young, 38% of the professionals with ages under 35 years old (considered career beginners), in comparison to 17% with 55 years old or more (close to retirement). Nevertheless, disparities between the regions were observed, with age ranges substantially older in the Region of the Americas (24% with 55 years old or more) and in Europe (approximately 18%), which represents an additional challenge for the replacement of these professionals^(^ 2 ^)^. In Brazil, the workforce can be considered young, with almost 35% of professionals aged less than 35 years old and 9% over 55 years old^(^ 2 ^-^ 3 ^)^. Regarding distribution by gender, nine out of 10 nursing professionals in the world are female. Important regional variations were found: 95% of the professionals in the Western Pacific Region and 76% in the African Region are women^(^ 2 ^)^. Data from 2017 indicate that, in Brazil, 87% of the professionals are female^(^ 2 ^-^ 3 ^)^.

In addition to the quantity data, a set of self-assessing questions was organized, aiming to allow for the identification of instruments and mechanisms in the dimensions: regulation, working conditions of nursing, and governance and leadership in the countries. In issues referring to the regulation of nursing education and practice (teaching institutions accreditation mechanisms, a national list of accredited teaching institutions, the existence of norms on education and content of the courses, norms for inter-professional education, norms of qualification of the faculty, the existence of an association of student nurses, evaluation based on competence for the practice, continuous professional development and advanced Nursing practices), more than 60% of the countries confirmed their existence, except for “advanced practice nurses” present in 53% of the responding countries and in 55% of the countries in the Americas. Brazil responded positively to seven of the nine questions, except for the advanced nursing practices and assessment based on competencies for the professional's practices (existing in 64% of the countries).

Regarding the working conditions (the existence of regulations on the working hours and conditions, regulation on social protection, regulation on minimum wage, measures to prevent violence against the health professionals, Nursing Councils to regulate on the nursing profession, and the existence of advanced nursing practices), more than 80% of the countries reported having regulations on working hours and conditions, social protection and minimum wage, and a Nursing Council or equivalent. Brazil responded positively to four of the six questions, except for the advanced nursing practices (included in the two question groups) and measures to prevent violence against the health professionals (37% of the countries reported having adopted measures to prevent violence).

Finally, on governance and leadership (the existence of a nursing leadership development program and a position in the government of senior/chief nurse), almost 71% of the countries reported having chief nurses in the government and 53% a nursing leadership development program. Brazil responded negatively to these two questions^(^ 2 ^-^ 3 ^)^.

Given this context and seeking to boost the contribution of nursing, valuing its competence scope of performance in the health inter-professional teams, the following is indispensable: adequate planning of the workforce, development of political interventions that enable the realignment of the nurses' training to the objectives of the health system, and optimization of investment to reduce the global shortage of these professionals^(^ 4 ^)^. These action lines are even more necessary given the challenges arising from the COVID-19 pandemic, which exposed the vulnerability of many health systems and, especially, the deficit of nursing professionals in the front line of the fight against the pandemic^(^ 5 ^)^.

Particularly for Brazil, the needs identified refer to broadening investment in the training of higher-level professionals, and in positions of political leadership occupied by nurses in the context of the elaboration and implementation of health public policies, situations that need to be tackled immediately.

Data also show the need for the development of public policies aiming at a better distribution of these professionals among the country's regions and federative units and points to big challenges such as the guarantee of adequate work conditions and environments, fair treatment and discrimination reduction, wage equalization and empowerment of the young professionals, and formulation of public policies sensible to gender differences. In this sense, it is suggested to foster the discussion among the several actors in their different levels, aiming at the implementation of evaluations based on competences for the professional practice and the definition of the performance scope of the “advanced practice nurse” in the country. Finally, the importance is reinforced of continuous investments in further analysis with a focus on the use of the number of vacancies offered and a terminal efficiency rate of the nursing courses, the market absorbing new the professionals, distribution of the nurses, technicians, and assistants among the care levels, municipalities and health regions and staffing of these professionals in remote and deprived rural zones, retention of the professionals in the services and in the health work market, and performance (productivity, response capacity, acceptance, accessibility), among others.

The data synthesized and presented in the State of the World's Nursing report and in the Picture of Brazil's Nursing infographic provide a current panorama of nursing and point to challenges for enhancing its work, highlighting the particularities of the profile of nursing in Brazil. It is hoped that they can be used as important information tools for decision-making processes and political dialog, in the projection and sustainability of nursing workforce agendas, allowing for the expansion of access to and coverage of the services, as well as strengthening the Unified Health System towards Universal Health. Finally, the importance of deepening the production of information and analyses by the Brazilian State emerges from the data, enabling the information of the political decision-making process, especially related to the management of the nursing workforce, in an increasingly challenging context like the one currently faced in Brazil and in the world.

## Footnotes

* In Brazil, there is a single Obstetrics undergraduate course, offered by the University of São Paulo (Bachelor in Obstetrics), authorized and recognized by the Higher Education Council of the State of São Paulo. The professional registry is made in the Nursing Regional Council, by virtue of a decision rendered in Public Civil Action No. 0021244-76.2012.403.6100, by the Federal Prosecution.

** Although there are 21 health professions and occupations regulated in Brazil, 14 of higher level and seven of technical level, the same professions considered in the global report were used for comparison purposes.
